# Sex-Based Difference in the Effect of Metoprolol on Heart Rate and Bradycardia in a Population-Based Setting

**DOI:** 10.3390/jpm12060870

**Published:** 2022-05-25

**Authors:** Linda C. Hendriksen, Grace Omes-Smit, Birgit C. P. Koch, M. Arfan Ikram, Bruno H. Stricker, Loes E. Visser

**Affiliations:** 1 Department of Epidemiology, Erasmus MC, University Medical Center Rotterdam, 3015 GD Rotterdam, The Netherlands; l.hendriksen@erasmusmc.nl (L.C.H.); grace.omessmit@outlook.com (G.O.-S.); m.a.ikram@erasmusmc.nl (M.A.I.); b.stricker@erasmusmc.nl (B.H.S.); 2 Department of Clinical Pharmacy, Tergooi MC, 1213 XZ Hilversum, The Netherlands; 3 Department of Hospital Pharmacy, Erasmus MC, University Medical Center Rotterdam, 3015 GD Rotterdam, The Netherlands; b.koch@erasmusmc.nl; 4 Department of Clinical Pharmacy, Haga Teaching Hospital, 2545 AA The Hague, The Netherlands

**Keywords:** sex differences, pharmacodynamics, pharmacoepidemiology, metoprolol, bradycardia

## Abstract

**Background:** Metoprolol, a beta-blocker, is used to reduce the heart rate. Although it has been demonstrated that the metoprolol plasma concentration is higher in women than in men, the same dose is recommended. In this study, we investigated whether the metoprolol concentration was associated with a stronger heart-rate reduction and bradycardia in women than in men. **Methods:** This study is part of the Rotterdam Study (RS), a population-based prospective cohort study. Blood samples from a random subset of 2000 participants were used to assess metoprolol plasma levels. An analysis of heart rate (beats per minute, bpm) and bradycardia (<60 bpm) was performed in metoprolol users with an ECG at the day of blood collection to study sex-specific differences in heart rate and the risk of bradycardia. **Results:** In total, 40 women and 39 men were included. There was a statistically significant association between metoprolol concentration and heart rate in women (*p*-value: 0.014) but not in men (*p*-value: 0.639). Furthermore, women in the highest concentration group had a more than 15-times-higher risk of bradycardia than women in the lowest concentration group (OR = 15.6; 95% CI = 1.1, 217.3); however, this was not seen in men (OR = 1.3; 95% CI = 0.1, 12.4). After adjustment for age, BMI, time between blood sample and ECG, hypertension, myocardial infarction, heart failure, atrial fibrillation, digoxin use, and calcium channel blocker use, the association between concentration and bradycardia in women remained statistically significant. **Conclusions:** Women, but not men, had a statistically significantly lower heart rate at higher metoprolol plasma concentration and a statistically significantly increased risk of bradycardia.

## 1. Introduction

Several frequently prescribed cardiovascular drugs are known to have a different magnitude of effect in men and women, including beta-blockers [1,2]. Metoprolol is a cardioselective beta-1 adrenergic receptor inhibitor indicated for the treatment of angina pectoris, heart failure, myocardial infarction, atrial fibrillation/flutter, and hypertension [3,4]. Adverse effects include fatigue, cold extremities, bronchospasm, and bradycardia [5].

One of the clinically relevant adverse cardiovascular effects of metoprolol is a too-strong reduction in heart rate (HR) or bradycardia as a result of blocking beta-1 receptors in the heart [6]. The magnitude of heart-rate reduction is directly correlated with the plasma concentrations of metoprolol [6,7,8]. Reductions in HR can be precisely evaluated by digitally measuring the RR interval, defined as the time elapsed between two consecutive R-waves of the QRS signal on the electrocardiogram (ECG) or the interval between successive heartbeats [9,10].

Women have been found to be 50–70% more likely than men to experience adverse drug reactions (ADRs), which may be caused by more frequent polypharmacy, increased drug exposure, and greater sensitivity to drug treatment [11,12]. Differences in body weight and composition; sex hormones; metabolism, for example, differences in CYP2D6 activity; and elimination can result in an increased drug exposure and have been causally associated with sex-specific drug effects [13]. A recent study based on half a century of globally collected individual case reports showed that approximately 60 percent of the total aggregated data referred to ADRs in women, versus 40 percent in men. The period associated with the highest risk for ADRs was observed in women after the onset of puberty (12–17 years of age) and especially around the reproductive age (18–44 years of age). While ADRs occurred more often in women, severe and fatal drug effects largely affected men [14].

Although there is evidence for sex-related differences in drug metabolism, efficacy, and safety of metoprolol, no amendments have been made to prescription guidelines [1,13,15,16]. Earlier, Luzier demonstrated that, after the daily intake of 200 mg metoprolol, women had a two-times higher Cmax than men [11]. According to a subsequent pharmacokinetic modeling and simulation study on dose equivalence in men and women, dose adjustments should be made based on sex (i.e., 100 mg for men vs. 50 mg for women) [17]. In addition, a large retrospective study of patients with heart failure showed that the effectiveness of metoprolol in women did not increase from 50% of the recommended dose [18].

However, these studies suggest that a sex-based difference of metoprolol on heart rate is explained by the difference in pharmacokinetics. To our knowledge, no prior studies have been performed to quantify the sex-specific heart rate in relation to the plasma concentration of metoprolol. Our objective was to test, in a population-based cohort study, the hypothesis that also the pharmacodynamic effects of metoprolol on the heart rate of women are stronger compared to men by exploring the association between the metoprolol plasma concentration and the heart rate and the risk of bradycardia.

## 2. Methods

### 2.1. Study Design, Setting, and Population

This study was performed within the Rotterdam Study (RS), a prospective population-based cohort study among people ≥45 years of age from the Ommoord district of Rotterdam in the Netherlands. At the start, in 1990, the inhabitants aged 55 years or older (*n* = 10,215) were invited to participate in the RS, and 78% of them agreed (RSI). In 2000, inhabitants who had reached the age of 55 years were invited to participate in the second cohort (RSII). Out of 4472 invitees, 3011 decided to participate. A third cohort (RSIII) started in 2006 and included 3932 inhabitants aged 45 years and over with the total study population of 14,926 participants by the end of 2008. They were all extensively examined at study entry as baseline and subsequent follow-up visits every 3 to 6 years. First, the participants were interviewed at home, and then they underwent an extensive set of examinations, e.g., ECG, echocardiography, CT-scanning, and MRI, with an emphasis on imaging (of heart, blood vessels, eyes, skeleton, and later brain) and on collecting bio-samples to enable further in-depth molecular and genetic analyses. The participants in the Rotterdam Study are followed for a variety of diseases that are frequently seen in an older population, including coronary heart disease, heart failure and stroke, and dementia, among several other chronic diseases. The complete medication records were available for almost all participants in the Rotterdam Study as of 1 January 1991 from all pharmacies serving the Ommoord region, with details on the product and international non-proprietary name, number of filled tablets/capsules, strength, prescribed daily dose, and duration of use. The Rotterdam Study has been approved by the Medical Ethics Committee of the Erasmus MC (registration number MEC 02.1015) and by the Dutch Ministry of Health, Welfare, and Sport (Population Screening Act WBO, license number 1071272-159521-PG). The complete design of the Rotterdam Study has been described in a separate publication [19]. A random sample (*n* = 2000) was taken to assess drug levels from the 4th cross-sectional round of the first cohort (RSI-4) and from the 2nd round of the second cohort (RSII-2).

### 2.2. Assessment of Heart Rate/Bradycardia

Bradycardia was defined as a heart rate in beats per minute (bpm) below 60. The heart rate was obtained via a 10 s 12-lead resting ECG performed on participants. Data were recorded by using an ACTA Gnosis IV ECG recorder (Esaote Biomedia, Florence, Italy) at a sampling frequency of 500 Hz and stored digitally. All ECGs were processed and analyzed offline by using the Standardized Modular ECG Analysis System (MEANS), a program that has been evaluated extensively. MEANS determines common onsets and offsets for all 12 leads together on one representative averaged beat, using template-matching techniques [20].

### 2.3. Assessment of Metoprolol Plasma Concentration

For a random sample of 2000 participants from the fourth follow-up visit, blood samples were drawn by venepuncture for the purpose of assessing drug concentration levels. Participants were considered as users if the calendar date of blood sampling fell within a prescription episode of metoprolol, calculated by dividing the total number of dispensed tablets by the prescribed daily number of tablets. Participants whose first prescription for metoprolol was dispensed within 90 days before blood sampling were considered as starters; all others were considered prevalent users. Of the participants who used metoprolol, plasma concentrations of metoprolol were analyzed with a validated rapid UPLC–MS/MS assay that was developed for the simultaneous analysis of several antiarrhythmic drugs [21]. A simple and fast sample preparation protocol with protein precipitation followed by ultra-performance liquid chromatography (UPLC) for chromatographic separation and mass spectrometric detection applying electrospray ionization (ESI+) and selected reaction monitoring mode (MS/MS) was used. Participants with a metoprolol plasma concentration below the Limit of Detection (LOD: 7 µg/L) were excluded. The metoprolol plasma levels were categorized in five categories (<20 µg/L [22], 20–50 µg/L, 50–100 µg/L, 100–150 µg/L, and ≥150 µg/L).

### 2.4. Confounders

The following clinical measures were determined at baseline and during follow-up visits at the RS research center: age (years), body mass index (BMI, kg/m^2^), and time delay between blood extraction and ECG (seconds, s). Additionally, indications for the use of metoprolol were also included, namely hypertension, defined as a systolic blood pressure >140 mmHg, diastolic blood pressure >90 mmHg, or use of blood pressure lowering medication [23]; history of myocardial infarction (MI), defined as self-reported MI at baseline or ECG abnormalities indicative of prior MI, atrial fibrillation (AF), and heart failure (HF), as diagnosed by a cardiologist or internist [20]; and diabetes, defined as having a fasting blood glucose level ≥7.0 mmol/L or use of glucose-lowering drugs, or self-report of diabetes as a comorbidity [24,25]. Moreover, digoxin and calcium channel blockers, which can cause bradycardia, were considered to be potential confounders.

### 2.5. Statistical Analysis

Only participants with an ECG and a metoprolol plasma concentration at the same day were included. Descriptive continuous variables are presented as mean ± standard deviation (SD). A linear regression analysis was used to examine the association between 

The metoprolol plasma concentration and the heart rate for both women and men. The association between the metoprolol plasma concentration and bradycardia (<60 bpm) was examined by using a logistic regression analysis stratified according to sex and adjusted for time between blood sample and ECG (s). Furthermore, a logistic regression analysis was performed while adjusting for time between blood sample and ECG (s), age (years), BMI at baseline (kg/m^2)^, history of hypertension, myocardial infarction, heart failure, atrial fibrillation, use of digoxin, and use of a calcium channel blocker. The logistic regression was used to calculate the *p*-value for trend. For all analyses, a 2-sided *p*-value of <0.05 was considered statistically significant. Multiple imputation was used to handle missing BMI values. Data were analyzed by using IBM SPSS Statistics (version 25.0. Armonk, NY, USA; IBM Corp.) and SAS Enterprise Guide (version 7.1. Cary, NC, USA: SAS Institute, Inc.).

## 3. Results

**Figure 1:**
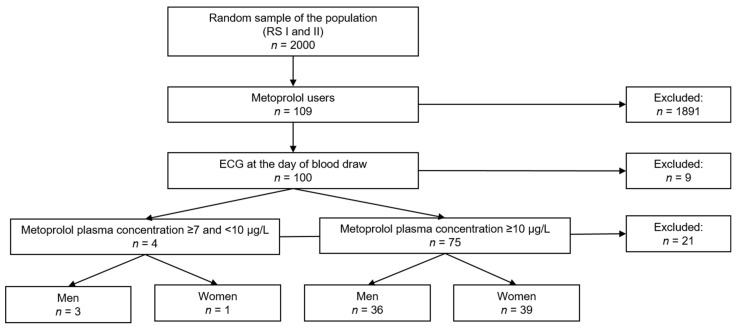
Flowchart of the inclusion of the study population.

Of the 2000 participants randomly selected for blood extraction, there were 109 metoprolol users, of whom 100 participants had an ECG measurement at the day of the blood extraction. Twenty-one participants were excluded because they had a metoprolol plasma concentration below the LOD. The study population consisted of four participants with a level <10 µg/L (lower limit of quantification) [21] and 75 participants with a metoprolol plasma level ≥10 µg/L (Figure 1).

The baseline characteristics of the study population are summarized in Table 1. The average age of the study population was 73.8 years (standard deviation, SD: 7.2), and the mean heart rate was 62.3 (SD: 9.4). In total, there were 12 women (30.0%) and 18 men (46.2%) with bradycardia (bpm < 60). There were no participants with AF, and, therefore, no adjustments for AF were performed in the logistic regression analyses.

### 3.1. Association between Heart Rate and Metoprolol Plasma Level

**Figure 2:**
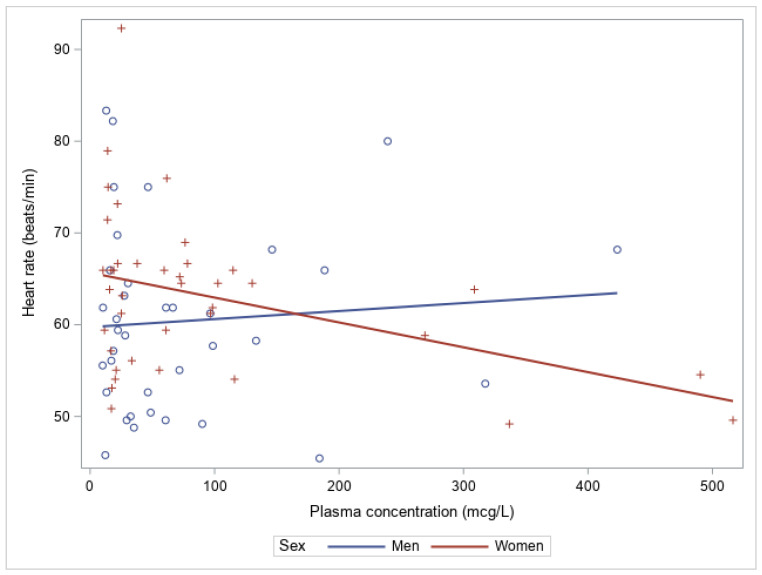
The association between the plasma concentration (µg/L) of metoprolol and the heart rate (beats/min) for men (blue circles; intercept, 59.735; slope, 0.009; 95% CI, [−0.029, 0.046]; *p*-value = 0.639; r^2^ = 0.006) and women (red plus signs; intercept, 65.665; slope, −0.027; 95% CI, [−0.048, −0.006]; *p*-value, 0.014; r^2^ = 0.153).

To assess the association between the heart rate and metoprolol plasma level, only the participants with a metoprolol plasma level ≥10 µg/L were included, due to the lower limit of quantification to ensure precise measurements. In women, a higher metoprolol plasma level was statistically significantly associated with a lower heart rate (*p*-value = 0.014; 95% CI = −0.048, −0.006) (Figure 2). However, in men, the heart rate was not statistically significantly correlated with metoprolol plasma level (*p*-value = 0.639; 95% CI = −0.029, 0.046) (Figure 2).

### 3.2. Association between Bradycardia and Metoprolol Plasma Level

To assess the association between bradycardia and metoprolol plasma level, the total study population was included, using five categories for the metoprolol plasma levels.

**Figure 3:**
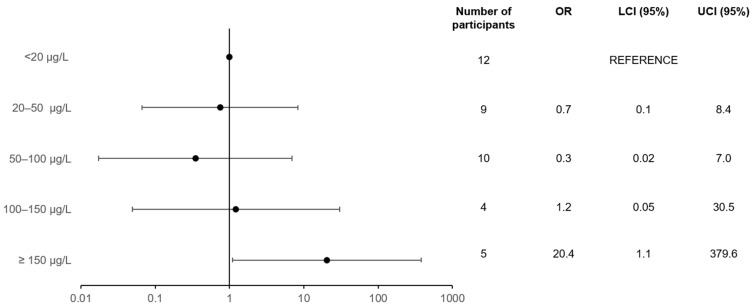
Association between metoprolol plasma concentration and the risk of bradycardia in women (*p*-value for trend: 0.08). Model: dependent = bradycardia (yes/no); independent = metoprolol plasma concentration (µg/L), time delay between blood draw and ECG (s), age at baseline, BMI at baseline, HT, HF, MI, digoxin use, and calcium channel blocker use.

Women with a metoprolol plasma level ≥150 µg/L had a statistically significantly higher risk of bradycardia than women with a metoprolol plasma level <20 µg/L (OR = 15.6; 95% CI = 1.1, 217.3, adjusted for time between blood sample and ECG). After further adjustment for age and BMI at baseline, the risk of bradycardia was still statistically significantly higher in the group of women with a metoprolol plasma level ≥150 µg/L (OR = 15.8; 95% CI = 1.1, 226.4) than the group with a level <20 µg/L. After further adjustment for the indications for the use of metoprolol and digoxin, as well as calcium channel blocker use, the risk of bradycardia in the group with the highest metoprolol plasma level remained statistically significantly higher compared to the group of women with the lowest metoprolol plasma level (OR = 20.4; 95% CI = 1.1, 379.6) (Figure 3).

**Figure 4:**
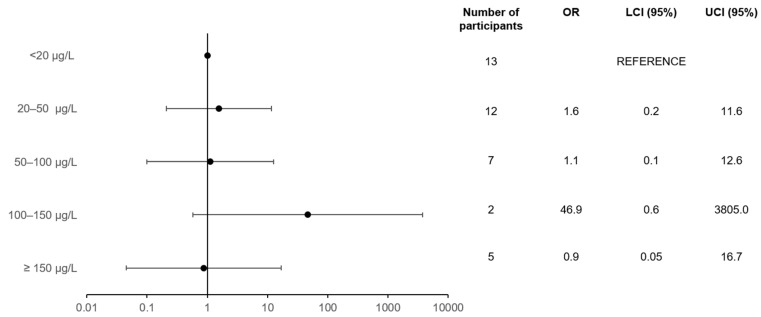
Association between metoprolol plasma concentration and the risk of bradycardia in men (*p*-value for trend: 0.7). Model: dependent = bradycardia (yes/no); independent = metoprolol plasma concentration (µg/L), time delay between blood draw and ECG (s), age at baseline, BMI at baseline, HT, HF, MI, digoxin use, and calcium channel blocker use.

There was no statistically significant difference in risks of bradycardia between the highest and lowest metoprolol plasma level groups in men (OR = 1.3; 95% CI = 0.1, 12.4, adjusted for time between blood sample and ECG). After further adjustment for age and BMI, there was no statistically significant difference in the risk of bradycardia between the highest and lowest metoprolol plasma level groups in men (OR = 0.9; 95% CI = 0.08, 9.8). Moreover, adjustment for all potential confounders showed no statistically significantly difference between the groups (OR = 0.9; 95% CI = 0.05, 16.7) (Figure 4).

For both men and women, adjusting for starting users did not change the results.

## 4. Discussion

In this population-based study, we demonstrated that women had a statistically significantly lower heart rate with increasing levels of metoprolol plasma concentrations, resulting in a higher risk of bradycardia in the group with the highest plasma level compared to the group with the lowest plasma level. The highest plasma levels were within or above the therapeutic range (20–340 mcg/L [22]) but below toxic concentrations (>750 mcg/L [22]). However, maximal beta-blockade is seen between 80 and 100 mcg/L [26], and concentrations above this range will cause the loss of beta-1 selectivity, resulting in more adverse drug reactions. In men, there was no association between metoprolol plasma concentration and bradycardia. After adjustment for time between blood sample and ECG, age, BMI, HT, MI, HF, calcium channel blocker, and digoxin use, the association between a higher metoprolol plasma level and higher risk of bradycardia in women remained. However, there was no statistically significant association between the metoprolol plasma concentration and bradycardia in men.

Our findings are in line with increasing scientific evidence which shows that women experience more adverse cardiovascular effects due to metoprolol than men [1,11,12,27,28]. Sex differences in drug-treatment response vary considerably because of disparities in drug pharmacokinetics (PK) and pharmacodynamics (PD) [1]. PK studies demonstrated that women have lower clearance of metoprolol, as well as a 50% higher maximum concentration and area under the curve (AUC), which make women more susceptible to ADRs [11,15,29]. Additionally, metoprolol exerted a greater pharmacodynamic response in women, resulting in larger reductions in systolic blood pressure and heart rate while exercising, likewise attributed to the PK difference in higher plasma drug concentrations [11]. This is in accordance with our results on the association between the metoprolol plasma concentration and heart rate and risk of bradycardia. Surprisingly, there was no association between metoprolol plasma concentration and heart rate in men. Prospective PK–PD studies could provide more information, since we had only a small sample of participants.

In the early trials where beta-blockers were tested for secondary prevention of myocardial infarction, women comprised only 20.5% of the study populations [30]. Despite the limited data from clinical trials on metoprolol in women resulting in the absence of dose-finding studies in women and the absence of revisions in official formularies worldwide, conventional prescribing habits are changing. Research conducted by our group revealed that women were prescribed lower starting doses of metoprolol than men, irrespective of current treatment guidelines [31]. Physicians from two independent data sources prescribed metoprolol, in both men and women, below the recommended doses and then up-titrating toward a well-tolerated dosage [31]. Furthermore, Santema et al. found that the optimal dose of metoprolol used in women with heart failure was 50% of the recommended dose in guidelines [18]. Moreover, in Sweden, a comprehensive web-based knowledge database (Janusmed Sex and Gender) was created to address awareness in sex-specific prescribing [32]. Progress in this domain may drastically lead the way to improvements in personalized medicine and ultimately decreasing the risk of adverse drug reactions in men and women in recently marketed drugs. However, the gap in knowledge of older drugs will remain unless prospective studies will be performed in the future.

Our study has several strengths and limitations. One of the strengths is the data source of a population-based cohort study, containing continuously collected participant information combined with medication data from pharmacy linkage and a long follow-up time. Owing to the robustness of our data, we were able to adjust for several variables (e.g., time interval between blood extraction and ECG, age, BMI, HT, MI, HF, digoxin, and calcium channel blocker use) that other studies would probably be unable to do. However, the main limitation of our study is that blood samples were collected only once, making it impossible to study changes in metoprolol plasma levels over time and only in a small number of participants. Furthermore, we had no information on the time between metoprolol administration and blood collection. The measured levels are assumed to be random levels and suggested to be normally distributed. In addition, a majority of the participants were chronic users, so this could result in the depletion of susceptibles, because the participants that tolerate metoprolol might be the ones with a lower risk of bradycardia. However, adjusting for starting users did not change the results. Another limitation is missing information on the adherence to the metoprolol treatment. The participants with detectable metoprolol levels used metoprolol; however, they could use less than prescribed. There are several studies that show that there might be sex and gender differences in adherence, but the evidence is still inconclusive [33,34,35,36].

In conclusion, we demonstrated that women had a statistically significant higher risk of bradycardia associated with a higher metoprolol plasma concentration. This remained after the adjustment for potential confounders. However, we did not see an association in men. These findings support prevailing data underlining the role of pharmacodynamic differences in the effectiveness and safety of metoprolol between women and men. Finally, this insight will help us move toward a safer use of metoprolol and personalized medicine in the future.

## Figures and Tables

**Table 1:** Baseline characteristics of the study population.

	Total Population	Men	Women	*p*-Value
	*n* = 79	*n* = 39	*n* = 40	
**Age in years, mean (SD)**	73.8 (7.2)	72.6 (6.6)	75.0 (7.7)	0.15
BMI, kg/m^2^ (SD) *	27.2 (3.6)	26.6 (2.7)	27.7 (4.2)	0.39
**Metoprolol plasma concentration, µg/L, mean (SD)**	79.3 (108.38)	70.3 (90.8)	88.1 (123.7)	0.57
**Metoprolol plasma concentration in categories**				0.97
<20 µg/L, *n* (%)	25 (31.7)	13 (33.3)	12 (30.0)	
20–50 µg/L, *n* (%)	21 (26.6)	12 (30.8)	9 (22.5)	
50–100 µg/L, *n* (%)	17 (21.5)	7 (18.0)	10 (25.0)	
100–150 µg/L, *n* (%)	6 (7.6)	2 (5.1)	4 (10.0)	
≥150 µg/L, *n* (%)	10 (12.7)	5 (12.8)	5 (12.5)	
**Heartrate in beats per minute, mean (SD)**	62.3 (9.4)	61.1 (10.1)	63.5 (8.7)	0.19
**Bradycardia, *n* (%)**	30 (38.0)	18 (46.2)	12 (30.0)	0.14
**Daily dose in mg, mean (SD)**	89.9 (50.9)	83.3 (51.4)	96.3 (50.2)	0.11
**Starter, *n* (%)**	3 (3.8)	1 (2.6)	2 (5.0)	0.57
**Hypertension, *n* (%)**	37 (46.8)	15 (38.5)	22 (55.0)	0.14
**Myocardial infarction, *n* (%)** ^#^	23 (29.1)	16 (41.0)	7 (17.5)	0.02
**Atrial fibrillation, *n* (%)**	0 (0)	0 (0)	0 (0)	
**Heart failure, *n* (%)**	8 (10.1)	4 (10.3)	4 (10.0)	0.97
**Use of digoxin, *n* (%)**	2 (2.5)	1 (2.6)	1 (2.5)	0.99
**Use of calcium channel blocker, *n* (%)**	25 (31.7)	12 (30.8)	13 (32.5)	0.87
**Diabetes Mellitus, *n* (%)**	11 (13.9)	6 (15.4)	5 (12.5)	0.71
**QT interval in ms, mean (SD)**	428.1 (31.0)	425.9 (32.0)	430.3 (30.1)	0.53
**Socioeconomic status**				0.12
Primary and low vocational, *n* (%)	37 (46.8)	13 (33.3)	24 (60.0)	
Intermediate, *n* (%)	25 (31.7)	15 (38.5)	10 (25.0)	
High, *n* (%)	15 (19.0)	10 (25.6)	5 (12.5)	
Missing, *n* (%)	2 (2.5)	1 (2.6)	1 (2.5)	
**Smoking status** ^$^				0.03
Never, *n* (%)	17 (21.5)	4 (10.3)	13 (32.5)	
Current, *n* (%)	12 (15.2)	4 (10.3)	8 (20.0)	
Former, *n* (%)	48 (60.8)	30 (76.9)	18 (45.0)	
Missing, *n* (%)	2 (2.5)	1 (2.6)	1 (2.5)	
**Alcohol consumption**				0.76
Non-drinkers, *n* (%)	13 (16.5)	7 (18.0)	6 (15.0)	
Drinkers, *n* (%)	63 (79.8)	30 (76.9)	33 (82.5)	
Missing, *n* (%)	3 (3.8)	2 (5.1)	1 (2.5)	

Abbreviations: SD—standard deviation, BMI—body mass index, µg/L—microgram per liter, ms—millisecond, mg—milligram. Continuous variables are presented as mean (+SD), and categorical variables are presented as count (percentage). * BMI missing for one man and one woman. ^#^ Statistically significantly different for men versus women (*p* = 0.0214). ^$^ Statistically significantly different for men versus women (*p* = 0.0282).

## Data Availability

The data that support the findings of this study are available from the Rotterdam Study, but restrictions apply to the availability of these data, which were used under license for the current study, and so are not publicly available. Data are, however, available from the authors upon reasonable request and with permission of the Rotterdam Study.
